# Developing a scalable training model in global mental health: pilot study of a video-assisted training Program for Generalist Clinicians in Rural Nepal

**DOI:** 10.1017/gmh.2017.4

**Published:** 2017-05-02

**Authors:** B. Acharya, J. Tenpa, M. Basnet, S. Hirachan, P. Rimal, N. Choudhury, P. Thapa, D. Citrin, S. Halliday, S. B. Swar, C. van Dyke, B. Gauchan, B. Sharma, E. Hung, M. Ekstrand

**Affiliations:** 1 Possible, Bayalpata Hospital, Sanfebagar-10, Achham, Nepal; 2 Department of Psychiatry, University of California, San Francisco, San Francisco, CA, USA; 3 Shared Minds, Boston, MA, USA; 4 Department of Psychiatry, B. P. Koirala Institute of Health Sciences, Dharan, Nepal; 5 Department of Psychiatry, University of Minnesota, Minneapolis, MN, USA; 6 Department of Anthropology, University of Washington, Seattle, WA, USA; 7 Henry M. Jackson School of International Studies, University of Washington, Seattle, WA, USA; 8 Department of Global Health, University of Washington, Seattle, WA, USA; 9 Department of Psychiatry, Kathmandu Medical College, Sinamangal, Kathmandu, Nepal; 10 Department of Medicine, University of California, San Francisco, San Francisco, CA, USA

**Keywords:** Mental health, Nepal, LMICs, Education, Training

## Abstract

**Background.:**

In low- and middle-income countries, mental health training often includes sending few generalist clinicians to specialist-led programs for several weeks. Our objective is to develop and test a video-assisted training model addressing the shortcomings of traditional programs that affect scalability: failing to train all clinicians, disrupting clinical services, and depending on specialists.

**Methods.:**

We implemented the program -video lectures and on-site skills training- for all clinicians at a rural Nepali hospital. We used Wilcoxon signed-rank tests to evaluate pre- and post-test change in knowledge (diagnostic criteria, differential diagnosis, and appropriate treatment). We used a series of ‘Yes’ or ‘No’ questions to assess attitudes about mental illness, and utilized exact McNemar's test to analyze the proportions of participants who held a specific belief before and after the training. We assessed acceptability and feasibility through key informant interviews and structured feedback.

**Results.:**

For each topic except depression, there was a statistically significant increase (Δ) in median scores on knowledge questionnaires: Acute Stress Reaction (Δ = 20, *p* = 0.03), Depression (Δ = 11, *p* = 0.12), Grief (Δ = 40, *p* < 0.01), Psychosis (Δ = 22, *p* = 0.01), and post-traumatic stress disorder (Δ = 20, *p* = 0.01). The training received high ratings; key informants shared examples and views about the training's positive impact and complementary nature of the program's components.

**Conclusion.:**

Video lectures and on-site skills training can address the limitations of a conventional training model while being acceptable, feasible, and impactful toward improving knowledge and attitudes of the participants.

## Background

In low-resource settings where access to mental health specialists like psychiatrists is severely limited, non-specialist clinicians often provide mental health services. This strategy, called ‘task-sharing’ (also called ‘task-shifting’), is a widely-accepted model to address the large gaps in the availability of mental health providers in low- and middle-income countries (LMICs) (Kakuma *et al.* 2011). Training and supporting generalists clinicians in mental health is a key step for successful task-sharing. The availability of simplified clinical protocols from the World Health Organization Mental Health Gap Action Program (mhGAP) has facilitated such efforts (World Health Organization, Mental Health Gap Action Programme and World Health Organization, 2010; World Health Organization, 2015).

Traditionally, training programs include specialist-led teaching, and occasionally, ongoing clinical supervision. In research studies that have provided evidence for mental health-related task-sharing in LMICs, all clinicians in the program received in-person training from specialists, including psychiatrists (Araya *et al.* 2003; Patel *et al.* 2010, 2003). Since psychiatrists are scarce in these settings, this model lacks scalability (Fairburn & Patel, 2014). In real-life settings, there rarely is dedicated funding, time, or human resources for psychiatrists to conduct full-length, on-site, in-person training for all clinicians.

A common training approach in LMICs is for select clinicians to travel from rural health centers to urban centers for several weeks of in-person training from specialists. This training strategy has three shortcomings. First, travel expenses limit the number of clinicians who can attend the training program. This will lead to lack of recognition of mental illness because most patients with mental illness do not present with a psychological symptom (Simon *et al.* 1999), but rather with non-specific aches and pains. Clinicians who have not received mental health training will routinely evaluate such patients leading to a large proportion of patients that may never receive mental health services, even though there is a trained clinician at the health center. Second, when clinicians leave for training, clinical services are often scaled-back or, in some situations, curtailed entirely in rural clinics. Third, given the high turnover of clinicians in rural sites, those with mental health training may suddenly depart. Without a scalable and readily available training program, new hires will have to wait until a training program is offered in urban centers.

In some settings, one of two strategies addresses these challenges. The first includes providing a ‘train-the-trainer’ package and asking the trained clinician to train the rest of the clinical staff at their rural site (Ray *et al.* 2012). This model is more scalable than traditional training, but its primary drawback is the uncertainty in fidelity and quality of the training provided by trainers at their respective sites. In the second strategy, a psychiatrist may be able to travel to rural clinics to provide on-site training. Such visits are usually shorter than the full-length training available in urban centers, and this model is still not scalable given the complete reliance on an in-person specialist. In addition, this challenge persists given lack of other mental health specialists, such as psychiatric nurses or psychologists (World Health Organization, 2011).

Internet-based and other technology-assisted strategies can provide scalable mental health training (Fairburn & Cooper, 2011). Internet training in cognitive behavioral therapy (Weingardt *et al.* 2009) and dialectical behavioral therapy (Dimeff *et al.* 2009) have produced similar results to in-person training. However, such studies have utilized personal electronic devices and a high-speed internet connection, both of which are rare in rural settings in LMICs. Organizations working in mental health in Nepal have utilized the various models described here from recruiting psychiatrists to train and supervise all clinicians (Lund *et al.* 2012) to conducting train-the-trainer workshops. Faced with these challenges, our team in Nepal developed an innovative training program that has the potential to be scalable in rural, low-resource settings. To our knowledge, our study is the first video-assisted mental health training program in this setting.

## Methods

### Setting

Nepal is one of the poorest countries in the world with a per capita Gross National Income of USD730 (The World Bank, 2014). Over 80% of Nepal's 30 million people live in rural regions (The World Bank, 2013), while its 54 psychiatrists are concentrated in a few major cities (World Health Organization, 2011; Luitel *et al.* 2015). The national health budget allocates 0.7% of its funds to mental health, the majority of which supports a standalone mental health hospital in Kathmandu (World Health Organization, 2011).

Our study site, Achham, is one of Nepal's poorest regions, ranking 73rd out of 75 districts, based on Human Development Index, and was severely affected by the 10-year Maoist War that ended in 2006 (Government of Nepal, National Planning Commission, 2014). Although 250 000 people live in Achham, access to specialists is severely limited. It takes 14 h by road to reach the nearest psychiatrist, and 12 h to a commercial airport. Our study site is a district-level hospital supported since 2008 by *Possible*, a non-profit health care organization, in partnership with the Nepali government. The 30-bed general hospital employs over 150 staff and has seen more than 350 000 patients since 2008. The outpatient primary care clinic, staffed by 27 physician and non-physician clinicians, serves about 300 patients a day.

### Participants

All clinicians employed at the hospital who directly provide care to patients with mental illness were included in the study. This included Community Medical Assistants (CMAs), who completed 15–18 months undergraduate training, including clinical rotations for 3 months; Health Assistants (HAs), who completed 36 months of undergraduate training, including 6 months of clinical rotations; Bachelor of Medicine, Bachelor of Surgery (MBBS) physicians, who completed 5 years of undergraduate training, including a 1-year clinical internship; and Psycho-social Counselors (PSCs), who completed a 6-month post-secondary training in counseling, psychosocial support, care coordination, and stress-reduction (Jordans *et al.* 2003). HAs, MBBS physicians, and PSCs evaluate patients in the outpatient clinic and the emergency department. CMAs evaluate patients in the emergency room. We invited all the clinicians present in the hospital on the days of the training to participate. Although this natural design resulted in variation of number of clinicians who participated in each training, all clinicians (*N* = 27) attended at least one training session.

### Ethics

The study was approved by the University of California San Francisco (#066667) and Nepal Health Research Council (#288/2014). Given that obtaining training and providing feedback is part of routine tasks, we obtained verbal consent, clarifying that we would not collect any identifying information. Clinicians were not required to participate in the study activities.

### Curriculum development

We designed a training program for the clinicians utilizing Kern's six-step process of curriculum development (Kern *et al.* 2009). In our prior study, we had conducted a needs assessment by organizing focus group discussions with 29 clinicians from three different hospitals in the region (Acharya *et al.* 2016*a*). We documented a substantial lack of mental health knowledge among physician and non-physician clinicians, and that clinicians sought additional training in mental health (Acharya *et al.* 2016*b*). Participants identified psychosis and depression as the most challenging mental illnesses to diagnose and manage. The clinical team recommended adding post-traumatic stress disorder (PTSD) to the curriculum given the anecdotally noted high rates of domestic violence and history of armed conflict in the region. Therefore, we selected depression, psychosis, and PTSD for the pilot curriculum. In addition, we selected two associated topics (grief to accompany depression, and acute stress reaction to accompany PTSD).

We developed learning objectives from the results of the needs assessment. Four bilingual, bicultural Nepali psychiatrists from the non-profit *Shared Minds* [BA, MB, SHi, and BS] discussed and refined these objectives and *Possible's* Medical Director [BG] approved them. The final list of learning objectives were tagged as knowledge, skills, and attitude-based competencies, and then divided into two categories: those that could be covered by a recorded lecture (mostly focused on knowledge and some on attitudes) and those that required in-person training (mostly focused on skills and some on attitudes).

The curricular design strategies included narrated video lectures and facilitated role-play vignettes. The psychiatrists from *Shared Minds* narrated slideshow-based lectures for the five priority topics. We used three primary sources to ensure that the material was evidence-based and relevant to a low-resource setting: two mhGAP protocols (World Health Organization, Mental Health Gap Action Programme and World Health Organization, 2010; World Health Organization, 2015) and *Where There is No Psychiatrist* (Patel, 2003). Because the target group for mhGAP protocols includes physician and non-physician clinicians, this allowed us to develop learning objectives that were appropriate for all participants. Given our earlier results that physicians rarely received any mental health training, we relied on the mhGAP protocols for all clinicians (Acharya *et al.* 2016*a*). For cross-cultural and linguistic adaptation we referred to validated psychiatric scales (Chen *et al.* 2013; Kohrt *et al.* 2016) and clinical protocols used in cross-cultural research studies in Nepal (Transcultural Psychological Organization Nepal, 2013), and included clinical vignettes drawn from our clinical experiences in Nepal.

Two clinicians, who were former employees at *Possible's* hospital and did not participate in the study, reviewed the lecture drafts. Those clinicians provided further cultural and contextual modifications (e.g. based on their recommendation, we edited the psychosis module to note that hearing voices in the context of traditional healing rituals was a normal experience and not a sign of pathology). When new terms were introduced (e.g. a literal translation of the term ‘depression’), we immediately included a simple language description for the word. This approach addresses some of the challenges in cross-cultural equivalence (Flaherty *et al.* 1988), especially in settings where English and local terms may not be technically and scientifically equivalent, and literal translations may not be recognizable to clinicians or patients. A common recommendation in such settings is to optimize equivalence, while still accepting that complete cross-cultural equivalence will likely never be achieved (i.e., no Nepali term will immediately capture all the cultural, social, medical, and conceptual meanings behind the term ‘depression’), and to develop additional explanations with the primary goal that the learners understand the concepts (Kirmayer & Swartz, 2014).

We then conducted narration for slide-based video lectures using conversational Nepali language and minimized the use of technical terms. This is in contrast to most health training in Nepal, where written protocols and didactic instruction heavily rely on English terms. We preferred using conversational Nepali to make the materials accessible to non-physician clinicians, whose English proficiency may be lower than that of physicians. We also intended to avoid situations where clinicians are forced to create their own translations when describing mental health concepts to patients and families. We used cultural and colloquial metaphors to describe certain concepts. For instance, the literal translation of PTSD is complex and foreign and has been found to be stigmatizing (Kohrt & Hruschka, 2010). To explain it, we used a common Nepali proverb *Agultaa le haneko kukur bijuli chamkida tarsincha* (‘A dog that was struck with burning firewood experiences extreme fear when it sees lightning’). This way, instead of memorizing an awkward, literal translation most likely unfamiliar to the patient, clinicians can also use the proverb to describe the ailment to patients and families.

On-site training included role-playing exercises based on cases developed by SS, who is a full-time psychiatrist in Nepal. This part of the curriculum built on the knowledge imparted by the recorded lecture. This approach allowed a more focused and shorter visit from the psychiatrist to the rural site, compared with a full-length training that would have covered all the curricular materials.

To avoid disrupting clinical services, clinicians viewed the five video lectures at the beginning of each day over a 5-day period. All clinicians gathered in a conference room, completed the pre-test, watched the lecture together, and completed the post-test immediately afterwards. On average, the entire process took approximately 1 h per topic. Three weeks later, a psychiatrist [SS] traveled to the hospital and provided on-site skills-building and clinical coaching to facilitate utilization of new knowledge in clinical practice. This part of the training lasted for a week and included three components: (1) Structured, simulated role-playing exercises for about 45 min before seeing real patients; (2) Demonstration of clinical interviewing, appropriate counseling and treatment planning with real patients; and (3) Coaching for clinicians by having the latter lead patient evaluations, while the psychiatrist provided guidance, asked probing questions to the clinician, and guided treatment planning. All trainees participated in role-playing exercises by breaking into groups of three, alternatively playing the roles of clinician, patient, and observer, with the psychiatrist providing feedback on role-playing. After conducting these practice sessions in the morning, the psychiatrist worked with clinicians in the afternoon in their care of patient, initially demonstrating interviewing technique and then observing and guiding the clinicians as they took the lead. We conducted the post-test about attitudes and the KIIs after the psychiatrist's visit. Following this training, the hospital decided to implement the collaborative care model, which includes the psychiatrist making weeklong visits to the hospital every quarter to reinforce clinical skills and remotely calling the PSCs every week to review all mental health cases (Raney, 2015; Acharya & Swar, 2016). This paper focuses on the development and assessment of the early, intensive mental health training.

### Data collection

#### Quantitative measures

Using Kirkpatrick's evaluation framework (Kirkpatrick & Kirkpatrick, 2006), the evaluation of the pilot curriculum had the primary goals of measuring: (1) learner satisfaction and (2) change in knowledge and attitudes regarding the identified topics, i.e., acute stress reaction, depression, grief, psychosis, and PTSD. A pre-test on the specific topic preceded each video lecture. Immediately after the video, the same questions were included in a post-test. This post-test also included questions on feedback of the video. The videos and the on-site training covered learning objectives regarding attitudes. We administered a pre-test for attitude change before the videos and again after the on-site training. Here we have provided details on instrument adaptation and development.

To assess learner satisfaction and acceptance, we included one question on immediate response to the training videos. Responses to this question were reported using a five-point Likert scale and included complete phrases in colloquial Nepali next to numerical ratings to improve validity (Krosnick, 1999). We also included free-form questions on what learners liked, did not like, and felt could be improved.

To assess application of knowledge, we recognized that existing testing instruments (e.g. from mhGAP) do not include case vignettes nor assess specific learning objectives for the topics identified by our team. In light of these limitations and the need to develop assessment tools based on specific learning objectives (Sanders *et al.* 2004), we developed instruments to assess learner knowledge using best practices (Haladyna *et al.* 2002). We also included a few negatively phrased questions to assess knowledge focused on avoiding harm in certain situations. For instance, in the test to assess knowledge of depression, we asked the learner to pick the case vignette where the clinicians should not prescribe an antidepressant. Most of the questions in the assessment tools were multiple-choice, and we extracted many plausible distractors from the results of the needs assessment focus groups (e.g. prescribing vitamins and analgesics rather than psychotropic medications for patients with mental illness). Two questions asked for free-form listings of depression and psychosis symptoms. For each question that assessed knowledge, we included a clinical vignette that was culturally and contextually relevant and tied to a specific learning objective for the topic. Participants received one point for each correct answer on the multiple-choice question and a point for a correct disorder criterion. We presented total scores in the pre- and post-tests as percentage points. We also asked participants to provide an overall rating for each video lecture out of a total score of five during the post-test.

Nepali psychiatrists [BA, MB, SHi, and BS] developed all questions and shared them with each other for feedback on relevance, validity, and readability. A non-physician researcher [PR] with training in evaluation methods reviewed each question for readability and acceptability.

To assess change in participants’ attitudes, we utilized questions from the mhGAP toolkit (not available publicly, but distributed to mhGAP trainers by the World Health Organization). We selected four out of 11 questions that were relevant to the five topics in our training program. Participants could choose ‘True’, ‘False’, or ‘I don't know’ for any given statement.

#### Qualitative measures

PR, who is a mental health researcher, conducted key informant interviews (KIIs) with eight generalist clinicians (*n* = 8) before and after the mental health training, on-site at the hospital. Each interview lasted 45–60 min. Leaders of the clinical team chose the participants based on interest in providing feedback, having participated in most of the training activities, and being representative of each category of clinician. We asked them about the overall experience, acceptability, benefits, and suggestions to improve the training. Although the sample size was based on parameters defined above, we did achieve saturation, as determined by lack of additional insights regarding the questions presented. We conducted the interviews in Nepali, audiotaped and transcribed them, and then translated into English.

### Data analysis

#### Quantitative analysis

We included participants who completed both the pre-test and post-test in the final analysis for each topic. Given the small sample size, we conducted two-tailed Wilcoxon signed rank tests to determine if there was a significant change in knowledge scores before and after participating in the online training for each topic. To assess attitudes about mental illness, we used a series of ‘Yes’ or ‘No’ questions and analyzed the proportions of participants who held a specific belief before and after the training by using exact McNemar's test. For the latter analysis, we used the categorical variables (‘Yes’ or ‘No’) rather than a percentage scoring system to maintain consistency with the mhGAP attitude questions, as noted above. To conduct all statistical analyses we used SAS 9.3 at a 5% level of significance.

### Qualitative analysis

We analyzed structured notes from the KIIs using an iterative approach to thematic analysis (Boyatzis, 1998), which resulted in a codebook with hierarchies and themes as described in the ‘Results’ below. PR initiated coding and BA reviewed the codebook against the notes. Both coders discussed any disagreements until they achieved consensus.

## Results

The number of participants who were available to complete both pre- and post-tests for various topics ranged from 13 to 19 because of leave, illness, and other reasons (Table 1). However, all clinicians available on the specific day of each training day participated.

**Table 1.** Characteristics of clinicians who completed at least one set of pre- and post-tests

Characteristics of participants	*n* (%)
Number of participants	27
Gender	
Female	2 (7)
Male	25 (93)
Professional degree	
Health Assistant	11 (41)
Bachelor in Medicine, Bachelor in Surgery	7 (26)
Community Medical Assistant	7 (26)
Psychosocial Counselors	2 (7)
Received any prior graduate mental health training	4 (15)
Years since graduation, mean ± s.d.	4.14 ± 2.99

### Reactions and feedback for video lectures

Generalists largely assigned high ratings (out of five) to each video lecture (Table 2) on a scale that ranged from ‘did not like it’, ‘liked it only a little bit’, ‘liked it moderately’, ‘liked it quite well’, and ‘loved it’. The most common positive comments in the free-form question were: ‘use of simple, conversational Nepali’, ‘use of mnemonics for symptoms’, and ‘inclusion of patient vignettes’. The most common comment under suggestions for improvement were: ‘no additional suggestions’, ‘include an interactive component’, and ‘utilize more case vignettes’. Of note, clinicians provided these reactions immediately after the video lectures and before the on-site skills-building training by the psychiatrist.

**Table 2.** Overall rating of video lectures using a five-point score

Module	*N*	Mean rating ± s.d. (total score 5)
Acute stress reaction	15	4.27 ± 0.70
Depression	19	4.53 ± 0.91
Grief	16	3.88 ± 1.15
Psychosis	13	4.08 ± 0.86
PTSD	13	3.92 ± 1.04

### Change in participants’ knowledge

There was a statistically significant (*p* < 0.05) increase in scores on knowledge questionnaires before and after the training for each topic except depression (Table 3). We noted the largest increase for grief, for which participants demonstrated a median 40 point increase in knowledge scores (*p* = 0.0001).

**Table 3.** Change in pre- and post-test scores on knowledge about each topic, total score 100 per topic

Module	*n*	Median score before training	Median change in percentage points (Δ)	Signed rank test statistic (*S*)	*p* value
Acute stress reaction	15	40	20	32	0.03
Depression	19	89	11	25.5	0.12
Grief	16	60	40	52.5	<0.01
Psychosis	13	67	22	30.5	0.01
PTSD	13	60	20	25	0.01

### Change in participants’ attitudes

We observed improvements in all four metrics adapted from the mhGAP toolkit on attitudes between the pre- and post-test (Table 4). We present data from 13 participants who were able to complete the pre-test, participate in the weeklong video-assisted training, the weeklong on-site training, and the post-test. In this sample, the number who believed that patients with mental disorders cannot make decisions about their health decreased from 7 to 1 after the training (*p* = 0. 0143). There was no statistically significant change in the attitude that patients receive the best care in mental hospitals. For the remaining two items, we did not conduct any statistical test because every single participant correctly noted that asking about suicidal thoughts does not increase the likelihood of suicide and that not all patients need to take medications.

**Table 4.** Change in attitudes towards mental health, *N* = 13

Statement: ‘Clinicians who believe that…’	Pre-test *n* (%)	Post-test *n* (%)	McNemar's test statistic (*S*)	*p* value
Patients with mental disorders cannot make decisions about their health	7 (54)	1 (8)	6.0	0.03
Patients with mental disorders are best cared for in mental hospitals	5 (38)	3 (23)	2.00	0.50
Asking people about suicidal thoughts increases the likelihood of suicide	3 (23)	0 (0)	^a^	^a^
All patients with mental illness must take medications	2 (15)	0 (0)	^a^	^a^

a No statistics computed since all participants selected ‘False’ for these two items in the post-test.

### Key informant interviews

Eight key informants participated in KIIs before and after the training. These included two HAs, two CMAs, three MBBS physicians and one PSC. There were no notable differences in these findings based on type of clinician. We have presented the results with exemplary quotes on acceptability, benefits, and suggested changes for the training.

#### Acceptability

All participants reported having an overall positive experience with the training. They noted that the video lectures provided a new experience and they had diverging responses. “*The videos were short, sweet and to the point,*” said one participant. However, some participants thought the videos were ‘one-way’ and suggested 10–15 min of interactive session immediately after each video presentation. Participants believed that the two components of the training were complementary because the videos provided background for the in-person training. Participants liked the role-plays embedded within in-person training: “*If we only hear we tend to forget, role plays have a deeper impact.*”

Some noted that traditional training that includes traveling to the city often provides a welcome break for clinicians in rural regions. However, they also noted that video-assisted training can help all clinicians.

### Benefits

Participants said that after the training they had a better understanding of mental illness, treatment, and medications. One said, “*Earlier, I used to avoid dealing with patients with mental illness, but now I am more prepared.*” Another participant said that prior to the training, clinicians only knew little bit about depression and psychosis, and did not know the various indications for prescribing psychotropic medications. One participant said that prior to the training, clinicians often misdiagnosed the patients: “*We did not try to find out their actual problems, some said ‘the body is possessed and you need to seek help from traditional faith healers.’ Others prescribed anti-depressants/anti-psychotics without proper diagnosis.*” Participants noted that the training has improved their clinical skills so they do not make such errors anymore.

### Suggested changes

Participants suggested increasing the frequency of training, including interactive sessions after each video and providing reference materials and books. Some participants sought more cases in the video lectures: “*The lectures did have examples but we could add more, selecting examples from the local context and hospital would be even better.*” Others said that on-site presence of a psychiatrist who can provide ongoing training would improve the mental health skills among clinicians.

## Discussion

Training non-specialists is a key goal in efforts to expand access to mental health services (World Health Organization, 2007; Collins *et al.* 2011; Becker & Kleinman, 2013). Prior efforts in developing core competencies among generalist clinicians to deliver mental health (World Health Organization, Mental Health Gap Action Programme and World Health Organization, 2010; Collins *et al.* 2015) require adaptation to local contexts. Traditional training methods utilizing classroom instructions face challenges of cost and access, while approaches using technology-assisted training are acceptable and feasible in various settings (Sweetland *et al.* 2016; Keynejad, 2016). This pilot study demonstrated that video-assisted training is acceptable to clinicians and can enhance competencies related to knowledge and attitudes regarding mental health. This provides an important proof-of-concept for a program that does not require a psychiatrist's presence for the full duration of the training. Focusing the psychiatrist's efforts on skills-based training provides a strategy to scale-up mental health training in low-resource settings. All the clinicians who were available on the day of the training participated, demonstrating high acceptability. Because we used a naturalistic design, there was variation in the participation rate among clinicians throughout the training. This may limit the feasibility of this intervention in reaching all the clinicians. However, it is important to note that this participation rate is higher than that for trainings in urban centers that only few clinicians can attend. In addition, training programs that attempt to reach all the clinicians risk disrupting clinical services. Finally, this hospital is able to show the video lectures for clinicians who may have missed the initial training, new hires, and those who seek refresher training.

Lack of a comparison group and the small size of both qualitative and quantitative samples are important limitations of the study. Because we used key informant interviews to obtain feedback from those who were most likely to have attended the trainings, there is a risk of bias towards positive response. Future studies can utilize focus groups with all participants. In addition, well-powered comparison studies can guide decisions to scale up such training programs against conventional models. Another limitation is that we administered pre- and post-tests for changes in knowledge competencies within an hour, and this could bias the results by not capturing long-term retention. We did not measure changes in clinician behavior or long-term retention of the positive changes in knowledge and attitudes. Positive attitude change could be affected by social desirability bias and these limitations can be remedied by follow-up studies that use standardized scales to monitor changes in clinician's behavior (Kohrt *et al.* 2015; Jordans *et al.* 2016). Data on knowledge retention, clinician behavior change, and clinical outcomes will be necessary to demonstrate long-term impact of the training and the subsequent interventions. Finally, we trained clinicians with varying levels of prior training and clinical roles. Even though the psychiatrist tailored the on-site training to the specific roles (e.g. PCPs focused on screening and prescribing, while PSCs focused on in-depth evaluations and psychotherapy), such differentiation will need to continue in follow-up trainings.

Some of our findings warrant further exploration. The lack of a statistically significant increase in knowledge for depression could have been due to a ceiling effect from the high scores in the pre-test, largely from correctly listing the criteria for diagnosing depression. In many health professional schools in Nepal, memorizing these criteria is often the only mental health training that students receive (Acharya *et al.* 2016*a*). This could have led to high baseline depression score, which is higher than any other pre-test score in the study. We found the largest increase in knowledge for grief. In this module, questions pertained to differentiating normal grief from psychosis or depression, and the importance of utilizing culturally-appropriate grieving rituals rather than immediately starting medications. It is possible that the lecture helped to correct clinicians’ attitudes towards medicalizing normal grief reactions. Finally, the only attitude change that did not achieve statistical significance was the belief that hospital-based treatment is the most appropriate for all mental illnesses. Although the training focused exclusively on outpatient treatment, it failed to explicitly mention that hospitalization is not the most preferable option for all patients. In the future, we can remedy this by emphasizing the importance of recovery and improved function in the community, rather than increased restriction by hospitalization for the majority of the patients.

Providing mental health training for every clinician is an essential component of global mental health initiatives that rely on task-sharing. We developed the study in response to practical challenges in providing mental health services in a rural region, and part of our larger efforts to build a delivery science platform in partnership with the Government of Nepal. Our approach has relevance in other LMIC settings by helping achieve an optimal balance between scalability, fidelity, and effectiveness of mental health training.
